# Parameterizing Toxic Stress in Early Childhood: Maternal Depression, Maltreatment, and HPA-Axis Variation in a Pilot Intervention Study

**DOI:** 10.1007/s11121-022-01366-4

**Published:** 2022-05-23

**Authors:** Rachael E. Wagner, Melissa Jonson-Reid, Brett Drake, Patricia L. Kohl, Laura Pons, Yi Zhang, Robert T. Fitzgerald, Mark L. Laudenslager, John N. Constantino

**Affiliations:** 1 Department of Psychiatry, Division of Child and Adolescent Psychiatry, Washington University School of Medicine in St. Louis, St. Louis, MO USA; 2 School of Social Work, Washington University in St. Louis, St. Louis, MO USA; 3 University of Colorado Anschutz Medical Campus, Denver, CO USA

**Keywords:** Toxic stress, Child development, Maltreatment, Resiliency

## Abstract

Adverse experiences superseding a child’s capacity to sustain regulation of emotion and adaptive function are theorized to constitute “toxic stressors” when they induce a deleterious biological response within an individual. We ascertained presumptive parameters of toxic stress among 164 low-income infants and toddlers (ages 4–48 months) from 132 families enrolled in Early Head Start (EHS). We randomized a subset of these families into a pilot intervention arm of parenting education (the Incredible Years, TIY), which supplemented the EHS curriculum. Official report child abuse and neglect (CAN) and child behavior were serially ascertained over the course of the study. We observed relatively low associations among maternal depression, CAN, caregiver-child relationship quality, hair cortisol, and adverse child behavioral outcomes. Moreover, despite poverty and the high prevalence (51%) of CAN in this sample, the frequency of clinical-level internalizing and externalizing behavior among the children did not exceed that of the general population, by their parents’ report. The pilot supplementation of EHS with TIY improved attendance in group meetings but did not significantly reduce adverse behavioral outcomes or CAN. This study revealed marked independence of standard indices of toxic stress (child maltreatment, maternal depression, caregiver emotional unavailability) which have been presumed to be risk factors for the development of psychopathology. That they were weakly inter-correlated, and only modestly predictive of child behavioral outcomes in this EHS sample, caution against presumptions about the toxicity of individual stressors, highlight the importance of ascertaining risk (and compensatory influences) comprehensively, suggest buffering effects of programs like EHS, and demonstrate the need for greater understanding of what parameterizes resilience in early childhood.

**Supplementary Information:**

The online version contains supplementary material available at 10.1007/s11121-022-01366-4.

## Introduction

The National Scientific Council on the Developing Child (NSCDC) developed the construct of *toxic stress*, defining it as “strong, frequent, or prolonged activation of the body’s stress response system” (National Scientific Council on the Developing Child, 2014), typically occurring in the absence of the buffering protection of a supportive caregiver (Shonkoff, 2016). Examples of stressors that may induce a toxic stress response include physical, sexual, or emotional abuse; chronic neglect; caregiver substance abuse or mental illness; exposure to violence; and/or the accumulated burdens of family economic hardship. It is theorized that these stressors are much more likely to evoke a pathological response of the stress response system if the child lacks buffering protection from adult caregivers, such as often occurs with maternal depression (Essex et al., 2002; Lupien et al., 2000, 2001). An essential characteristic of this phenomenon is a deleterious *biological* response to a stressor during sensitive developmental periods. According to the NSCDC, potential biological consequences include the disruption of developing brain structure and function; changes in gene expression; and a lowered threshold for stress system activation, which can lead to greater risk of physical diseases, psychopathology, and cognitive impairment - not only during childhood and adolescence, but also into adulthood (Jonson-Reid et al., 2010, 2012; Lanier et al., 2010).

Knowledge about what constitutes “toxic” stress, i.e., *excessive* activation of the body’s stress response system has been inferred indirectly from (i) epidemiologic associations between adverse life events and child outcome; (ii) laboratory assays of biomarkers during stress states among volunteer enrollees, recently including neuroimaging and psychophysiologic assessments; and (iii) studies of animal models. In this study, we examined associations between putative toxic stressors and outcome *within* a cohort of children and families who met stringent standards for poverty by virtue of eligibility and enrollment in the principal U.S. intervention program to support low-income infants and toddlers and their families, Early Head Start (EHS). The exclusive focus on a sample homogeneous with respect to poverty affords unique opportunity to interrogate presumed associations between predictors and outcomes within the context of substantial elevation in stress, to parameterize resilience, and to identify “exceptions” to assumed rules and roles of toxic stress in early childhood development.”

The last two decades of research on the deleterious effects of adverse early life experiences have generally supported a stress-diathesis conceptualization, in which (a) individuals vary in their capacity to mount an adaptive response to a given stressor; and (b) higher-acuity stressors - at the extreme of typical environmental variation - are much more likely than lower-acuity stressors to adversely affect development (Constantino, 2018; Shonkoff et al., 2012). However, it is difficult to know whether a given environmental stressor is exceeding a young child’s capacity to manage it without lasting brain or behavioral compromise. For this reason, biological markers - particularly those which can be assayed early in life - have been invoked as key indices of a pathological response to stress.

One such biomarker that has received a substantial amount of investigation is the stress hormone, cortisol. Levels of cortisol in blood, urine, and saliva have historically been used to index activation of the hypothalamic–pituitary–adrenal (HPA axis in response to stress (Hellhammer et al., 2008; McCallister et al., 2004; Papadimitriou & Priftis, 2009). However, cross-sectional measurements using these biomarkers have been inconsistent in their respective associations with carefully measured environmental stressors and concomitant profiles of behavior (Adam & Kumari, 2009; Gunnar et al., 2009). One possible reason for these inconsistencies relates to the dynamic nature of cortisol release and the possibility that cross-sectional assays at single points in time do not adequately reflect chronic perturbations of the HPA axis (Adam & Kumari, 2009; Segerstrom et al., 2004; Trickett et al., 2010). Prior studies in non-human primates have suggested that hair cortisol concentration (HCC) levels might reliably assess HPA activity over an extended period and provide greater predictive power in determining whether environmental challenges are chronically exceeding an individual’s adaptive capacity (Fairbanks et al., 2011).

When considering the types of stressful life experience that can be presumed to be “toxic” to children, poverty is often invoked (and is a particular focus of this study), but a more salient and direct stressor is child maltreatment, to date the most influential and modifiable (i.e., non-genetic) *cause* of developmental psychopathology in childhood.. Child maltreatment disproportionately affects low-income populations (Mersky et al., 2021), yet its proper ascertainment is challenging. Retrospective measures have the potential to be affected by recall and response bias (Widom et al., 2004), but they capture a greater number of individuals affected by maltreatment than prospective measures (Sedlak et al., 2010). In contrast, prospective measures, such as official reports retrieved from State administrative records, are regarded as having more validity (i.e., specificity), and may tend to capture the more severe cases, but there are also limitations to official report; it has been determined that prospective and retrospective measures of child maltreatment identify non-overlapping populations of children (Baldwin et al., 2019. In this study, we collected official report data (i.e., a prospective measure) from the States of Missouri and Illinois to assemble a rare co-registration of valid ascertainment of child maltreatment with measures of HPA-axis variation, maternal depression, and parent–child relationship characteristics in examining their respective associations with behavioral outcomes of young children in poverty.

Critically, there is evidence that the effects of even the most severe psychosocial stressors may be mitigated by means of interventions that provide supportive caregiving, nurturing, and engagement (National Scientific Council on the Developing Child, 2014; Shonkoff, 2016). Early Head Start (EHS) is a federal program which provides comprehensive support services to families of infants and young children who are at or below the national poverty line, a risk factor associated with a number of presumed toxic stressors. This study involved families enrolled in EHS, a subset of which received enhanced parenting education using the Incredible Years (TIY) curriculum. Substantial work has been done to establish the efficacy and effectiveness of TIY, an intervention which seeks to decrease harsh, critical parenting and cultivate supportive and responsive caregiving, elements essential for children’s emotional, social and behavioral development (Barlow, 2007; Foster et al., 2007; Gardner et al., 2006). There is also evidence supporting the effectiveness of TIY for high-risk populations, such as among families involved in the child welfare system (Linares et al., 2006) and in particular EHS families (Gross et al., 2003; Hurlburt et al., 2013).

We undertook this multi-stage study with the following goals: (1) to validate HCC as a biomarker of HPA-axis variation; (2) to explore the associations between a key set of presumptive toxic stressors with one another and with behavioral outcomes of children in poverty; and (3) to pilot the supplementation of the EHS program with an a structured, evidence-based parenting education curriculum. We hypothesized that theorized risk factors for adverse child outcomes, ones which have been presumed to elicit a toxic stress response - poverty, maternal depression, deficits in emotional availability of parents, and CAN - would be associated with one another, HPA axis abnormality, and behavioral deviation in early childhood. Further, we predicted that their effects might be buffered by enhancing parent education via TIY.

## Methods

**Fig. 1:**
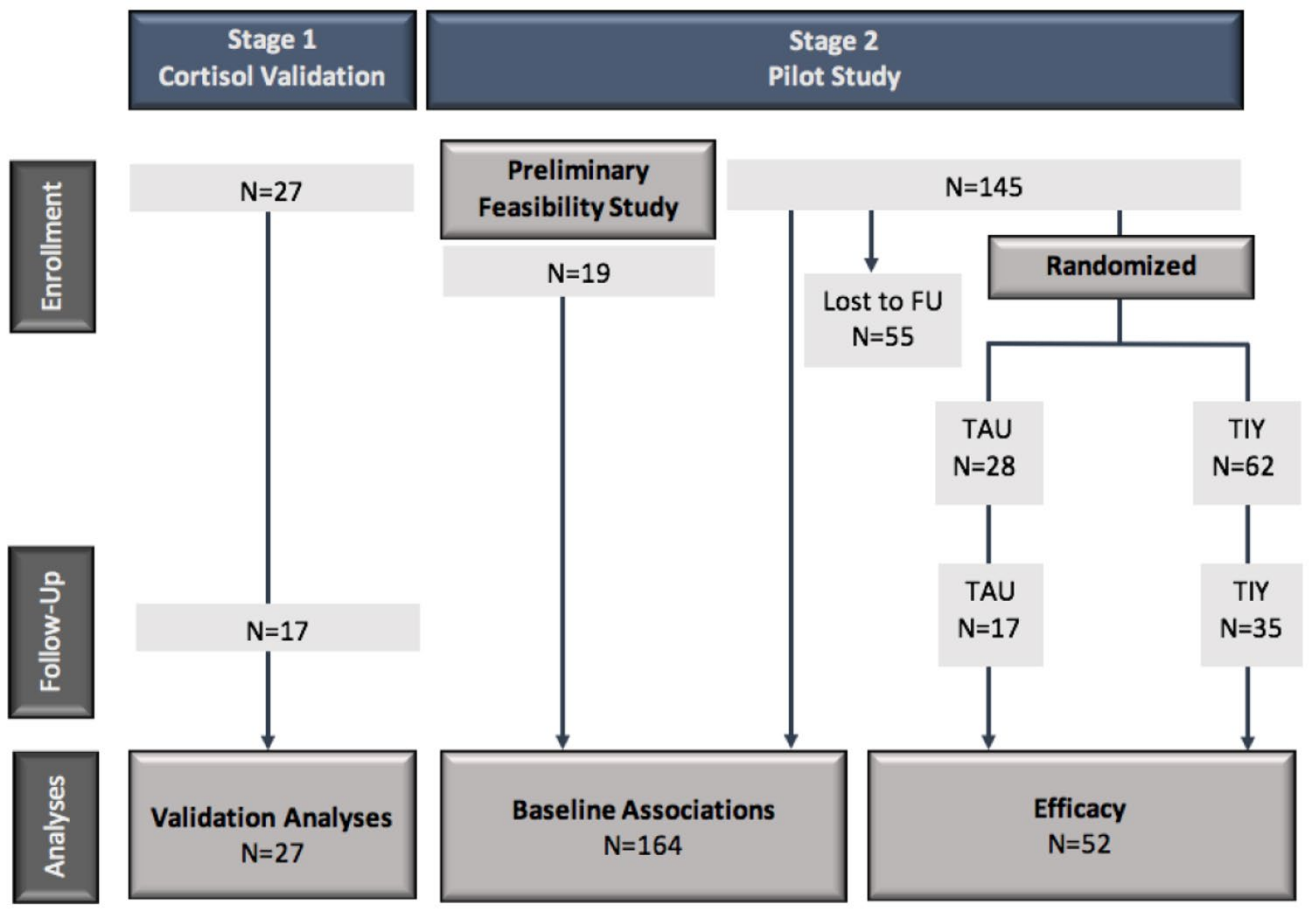
Study design and subject flow from enrollment to analysis. For families randomized to TIY versus TAU, randomization occurred at the level of *family educator*; all participating clients of a given EHS family educator (usually 2–5 clients at any given time) were assigned as a cluster to TIY versus TAU on the basis of random assignment of their respective family educator to one or the other condition. EHS family educators with participating (enrolled) families were randomized in sets of three, 2:1 TIY versus TAU, after the EHS clients in each of their respective caseloads had had the opportunity to consider participation and at least one completed the informed consent process. FU, follow-up; TAU, treatment as usual; TIY, The Incredible Years

The study design was divided into two stages, as depicted in Fig. 1: In a first stage, hair cortisol was validatedas an index of HPA-axis variation. Subsequently, we proceeded with a pilot intervention study of risk and outcome *within* EHS, which included a randomized controlled trial arm; all Stage 2 families were enrolled in EHS.

### Stage 1: Validation of Hair Cortisol Concentration Assays

#### Subjects

We recruited 27 subjects (9 female) in the greater St. Louis, Missouri area from local early childhood centers and collaboration with a local research study, Early Childhood Connections (ECC; R34MH083871). This sample of children was strongly enriched for poverty and reported child maltreatment (Jonson-Reid et al., 2018) - the sub group who enrolled in this validation study had a mean age of 34.37 months (SD 12.15, range 10–58 months); racial distribution was 40.7% African American, 33.3% Caucasian, and 25.9% other.

#### Procedure

We collected samples of overnight urine, hair, and saliva from the subjects. First, urine samples were obtained from an overnight collection. The following morning, saliva samples were obtained. Parents were instructed to contact the research team immediately upon the child’s awakening. The team members were stationed in a public location near each respective family’s home starting one hour prior to the child’s usual wake-up time, so that the initial saliva collection could be captured precisely 30 min after awakening. Thus, in this meticulously coordinated real-world collection (in a high-risk sample), the saliva acquisitions occurred on average, 32 min (SD = 5.7 min) after the child awoke. Saliva was collected again at 4 pm on the same day. This procedure was repeated at three-month follow-up (T2) for 25 of the 27 original subjects. Following the afternoon saliva collection, and per published protocols (Hoffman et al., 2017), approximately 50 strands of hair were snipped at the base of the hair shaft from the posterior vertex. The first three centimeters of hair length from the base were assayed for hair cortisol concentration (HCC).

Details of the laboratory assays of cortisol from the respective biomaterials are provided in Supplementary Materials.

Continuous heart rate recordings were obtained from the children over the hours between the first and second saliva acquisition using a Polar rs400 heart rate monitor applied to the child’s chest on the day of each biomaterials collection. Resting heart rate variability was calculated over intervals in which the child’s heart rate was less than 140 beats per minute.

#### Stage 1 Data Analysis

HCC was log transformed to approximate a normal distribution. HCC outliers were removed using standard interquartile range (IQR) formulations (1.5 × the IQR) after the log transformation. The skewness and kurtosis of the non-log-transformed data were 7.81 and 68.8, respectively; after transformation, skewness was 0.84 and kurtosis was 0.626. Pearson’s correlation coefficients were computed for bivariate associations among the variables, and intra-class correlation (ICC) coefficients were computed to estimate each measure’s temporal stability.

### Stage 2: Risk and Outcome in Early Head Start

#### 2A. Initial Feasibility Sample

In order to ensure the feasibility of implementing TIY within an EHS curriculum, we enrolled 13 families from a semi-rural EHS program in the Eastern third of Missouri, comprising 19 children aged 12–48 months. We supplemented their families’ biweekly group visits (see below) with TIY for a six-month period. After completion of TIY, fmilies returned to the usual schedule of socialization visits. Curriculum was administered by two trained and certified TIY group leaders under the employment of Washington University School of Medicine (WUSM). These individuals completed all measures to ensure fidelity to TIY model.

#### 2B. Risk and Outcome in a Randomized Controlled Trial

##### Subjects

Having established the feasibility of our study design, we recruited 145 subjects from 125 families enrolled in Early Head Start programs in Warren County, Missouri and Madison County, Illinois, over calendar years 2015–2017. Both sites participated in EHS using the Home-Based program option, in which the Parents as Teachers (PAT) home visitation model and Creative Curriculum Teaching Strategies served as the core curricula in use by family educators during weekly home visits. Consistent with the EHS Program Standards for the Home-Based program option, biweekly group meetings were a core component of the EHS intervention (see below). At the time of their respective enrollments, the children’s ages ranged from 5 to 48 months. Primary caregiver ages ranged from 17 to 45 years; in our sample, all but four primary caregivers were mothers; two primary caregivers were fathers; one was an aunt; and one was a grandmother. Following enrollment and baseline data collection, 55 children were lost to follow up on the basis of moving out of the service area or lack of response to research contacts. The remaining 90 children were randomized by family educator (see below) into supplemental group-based parenting education using the Incredible Years curriculum. Of these, 52 were able to be reached by the research team 6 months following initial assessment. Descriptive statistics for this group and the entire study sample are provided in Table 2.

**Table 1:**
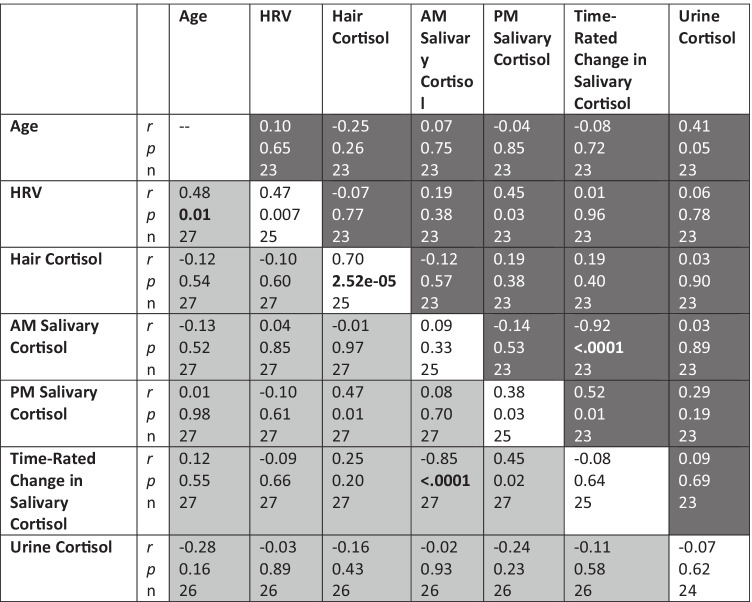
Correlation and ICC matrix of HPA-axis biomarkers

Pearson’s coefficients of correlation between the various measurements at baseline (light gray) and at three-month follow-up (dark gray). Time 1 to Time 2 ICC for each measure are presented along the diagonal. Time-rated change in SCC is the change in SCC from morning to afternoon, divided by the time in hours. Statistically significant correlations (*p* < .01, adjusted for multiple comparisons using a Bonferroni correction) are in bold

**Table 2:** Descriptive summary statistics of the sample

**Variable**	**Number of subjects**	**Mean (SD)**	**Number of subjects**	**Mean (SD)**
	**(full sample)**		**(sub group followed at 6 months, *n* = 52)**	
**Region**		-		
Rural	123		40	
Urban	39		12	
Missing data	2			
**Race**		-		
White	99		34	
Black	31		8	
Other	34			
			10	
**Ethnicity**				
Hispanic	16		7	
Non-Hispance	141		45	
Unknown	7			
**Caregiver Level of Education**		-		
No HS grad				
Grad or GED	26		12	
Some post-HS	52		21	
	58		12	
**Caregiver Marital Status**		-		
Single	56		14	
Married	41		18	
Living with partner	28		11	
Divorced	6		2	
Separated	5		0	
**Sex**		-		
Female	82		28	
Male	82		24	
**Caregiver CES-D**		13.17 (9.60)		13.23 (9.60)
Clinical^a^	45		16	
Normal	89		28	
**CBCL: Internalizing**		46.63 (10.61)		46.80 (10.56)
Normal	83		33	
Clinical^b^	4		2	
**CBCL: Externalizing**		47.74 (10.36)		46.06 (9.70)
Normal	81		33	
Clinical^b^	6		2	
**CCSERRS**	138	16.98 (4.76)	50	17.64 (4.42)
**Hair cortisol concentration**^c^	135	1.64 (.67) pg/mg		1.76 (.62) pg/mg
			48	
**Salivary cortisol concentration**	146	0.15 (.24) ug/mg	49	.15 (.19) pg/mg
**CAN**^**d**^		-		
** No-CAN**	59		19	
** CAN**	61		25	

Table 2 provides subject counts for demographic variables and all measures employed in the study, as well as mean and standard deviation (SD) for relevant variables. Unless otherwise indicated, variables reflect child-level data

^a^Clinical cutoff for CES-D utilized in this sample was ≥ 16

^b^Clinical cut-off for CBCL-Internalizing and Externalizing was ≥ 64

^c^Log transformed values

^d^CAN status is operationally defined as an official Child Protective Services report for any child in a family

##### Randomization

EHS families were randomized 2:1 to supplementation with (TIY) curriculum *according to EHS family educator*, following determination of study eligibility and the obtaining of consent. Families were informed that they would be assigned to a supplemental education track (TIY) or control condition on the basis of whether their assigned EHS family educator was randomized to one or the other condition. During the study years, the family educators carried caseloads of 2–5 EHS families as well as 5–10 families enrolled in Head Start (HS), to ensure continuity of support when children age out of EHS at age 3. Randomization was conducted at the level of family educator in order to minimize “contamination effects” (i.e., knowledge gained by family educators through co-participation with their clients in TIY being transferred to families assigned to the control condition over the 6 months of biweekly intervention). This arrangement, an application of a cluster-randomized-trial design (Cook et al., 2016), also afforded alignment of the intervention trial with the programmatically embedded continuity of relationships between family educators and their respective clients as EHS children cross the threshold of eligibility for HS services at age 3. Specifically, families were recruited into the study through advertising flyers and outreach communications circulated by the participating EHS programs and their family educators. After all EHS families of a given family educator had had a chance to consider participation in the study and made the decision whether or not to enroll, those who did enroll comprised a cluster (identified by family educator) in the randomization procedure. For a given set of 3 family educators, each with a “cluster” of one or more participating clients, a random number generator was used to assign a code to each of the three family educators; the cluster of participants who were clients of the family educator with the lowest numeric value for the assigned code were designated as control subjects. The participants were informed of their schedule of participation (intervention protocol versus control protocol) on the basis of the designation of their family educator (see Fig. 1).

##### Implementation of The Incredible Years

TIY was delivered in five waves, each of 6 months duration, by two group leaders. Each month, as a core element of the EHS curriculum itself, all families are encouraged to participate in one socialization group meeting and one education group meeting on an alternating biweekly schedule. Socialization groups serve to expose both parents and children to developmentally appropriate learning opportunities in a group setting, and to provide social opportunities for parents who are often isolated in their role of caring for young children. For families randomized to TIY, the usual curriculum for biweekly EHS socialization group meetings was substituted with TIY curriculum over a period of 6 consecutive months (12 manualized meetings of TIY), with childcare services provided to afford parents opportunity to fully engage and participate in the sessions according to the IY protocol. After completion of TIY, families resumed the usual schedule of EHS group meetings. In both the intervention and control groups, families continued to receive weekly home visits from their family educators before, during, and after the intervention period.

##### The Incredible Years Curriculum

The Incredible Years (TIY) differs from the curricular content of EHS group meetings in that it is a structured, evidence-based program of parenting education in which video vignettes, role play scenarios and active discussion of foundations of developmentally appropriate parent–child interaction are discussed with trained group leaders and among participating parents. The studies which comprise the evidence base for the effectiveness of TIY (as cited in 1ntroduction) have emphasized the importance of systematic use of praise, active listening and descriptive commentary, and the ignoring negative of behaviors in shaping children’s social and emotional development in the context of parenting. These are techniques that are taught by group leaders and mastered by parents over the course of the curriculum. We hypothesized that completion of the curriculum by parents would enhance parents’ roles as bufferers of the toxic effects of poverty, increase sensitive-responsive parenting and increase parents’ vigilance in preventing their children from encountering abusive or neglectful experiences.

In summary, following enrollment of age-eligible EHS children residing within a given service area, clusters of enrolled children sharing a given family educator were identified, sets of three comparably sized clusters were defined (each assigned to their own family educator) and two of the three clusters (categorized by family educator) were selected at random for assignment to the intervention condition. The intervention group adopted a separate location for group visits on the same schedule as their control counterparts, and the standard EHS group meeting content (for alternating socialization and education group visits) was replaced with TIY for 6 consecutive months.

##### Outcomes Monitoring

Data were collected at enrollment and at the end of TIY intervention period (6 months following baseline). Subjects were evaluated on pencil-and-paper measures completed by the child’s primary caregiver, clinician observation, and cross-referencing with State administrative records for official report child maltreatment

##### Measures

###### Hair Cortisol Concentrations (HCC)

HCC was assayed as outlined above in Stage 1.

###### Salivary Cortisol Concentrations (SCC)

SCC was assayed as outlined above in Stage 1.

###### Official Report Child Abuse and Neglect (CAN)

As a condition of participation in this research program, families consented to cross-referencing their identifying information and all research data with official state administrative records, including reports of CAN during and after the pilot intervention. All of the research information except the identifiers was encrypted. The encrypted information was submitted with identifiers to the Department of Social Services (Missouri) and the Department of Health Services (Illinois), where it was cross-referenced with official report records at an individual level. The linked dataset was subsequently stripped of individual identifiers before being returned to the research team. Official reports included placement in foster care (FC), substantiated reports of maltreatment (SRM), and unsubstantiated reports of maltreatment (URM). It should be emphasized that according to the harm/evidence model of substantiation, an SRM is not equivalent to verifying the presence of maltreatment, but rather a label used when sufficient evidence and/or risk of harm exists to permit family court intervention if needed (Drake & Jonson-Reid, 2000). In Missouri, both SRM and URM have been eligible to receive in-home or FC intervention for many years. It was not possible to specify which child or how many children in a family were subjected to maltreatment; nor could we ascertain the age at which maltreatment first occurred, since (a) date of first available report is not the same as the time when maltreatment began; and (b) some of the reports represented recurrences of unsubstantiated maltreatment have been purged. However, we note that abuse and neglect likely affect all children in a family.

###### Center for Epidemiological Studies Depression Scale (CES-D)

The CES-D is a widely utilized depression screener (20 items), with established reliability and validity across a wide variety of health conditions and demographics, both nationally and internationally (Radloff, 1977). The instrument was used to ascertain the following maternal depressive symptoms by self-report: depressed mood, feelings of guilt and worthlessness, feelings of helplessness and hopelessness, psychomotor retardation, loss of appetite, and sleep disturbance.

###### Caregiver-Child Social Emotional Rating Scale (CCSERRS)

The CCSERS is designed to assess the quality of caregiver–child socioemotional interactions and relationships (McCall et al., 2010). Characterized by established validity and reliability, this clinician-observation scale examines the caregiver’s emotional availability, affect of child and caregiver, the mutual nature of caregiver-child interactions, and the caregiver’s responsivity to the child’s lead. Parents and their children were directly observed in free play at baseline and follow-up by trained clinicians who completed the 15-min assessment at each time point.

###### Child Behavior Checklist (CBCL)

The CBCL (i.e., Achenbach Scales of Empirically Based Assessment, Achenbach & Rescorla, 2000) is parent- and/or teacher-report instrument assessing emotional and behavioral problems during preschool and childhood. The widely-used CBCL has undergone extensive establishment of its validity, reliability, and psychometric properties (Verhulst & van der Ende, 1992, 1995). The various syndrome scores of the CBCL can be combined to form Internalizing and Externalizing composites, which we utilized as outcome measures of child psychopathology.

##### Stage 2 Data Analysis

During preliminary analyses, we log transformed HCC to approximate a normal distribution. We then conducted two sets of primary analyses in order to examine two lines of questioning: (1) baseline data analysis and (2) baseline-to-follow-up analysis. In our baseline analyses, we tested associations among quantitative measures in a correlation matrix, conducted *t*-tests to compare measures as a function of the presence or absence of CAN, and performed linear regressions to determine if theoretically important variables - maternal depression and CAN - predicted our dependent variables: internalizing and externalizing child psychopathology. We utilized a repeated-measures ANOVA to determine the presence of an intervention effect by ascertaining if scores on the pre- and post-measures changed significantly between baseline and follow-up, for both TIY and TAU groups. Given that CAN and HCC were explored as both predictors of child behavioral outcome and as intermediate effects of intervention, we adopted a conservative approach to the correction of statistical significance using Bonferroni methods. The data analysis for this paper was generated using SAS/STAT, Version 9.4 of the SAS System for Microsoft Windows Copyright © 2002–2012. SAS Institute Inc. SAS and all other SAS Institute Inc. product or service names are registered trademarks or trademarks of SAS Institute Inc., Cary, NC, USA.

##### Compliance with Ethical Standards

Conducted at Washington University School of in St. Louis as a collaboration by investigators at the School of Medicine, Department of Psychiatry, and the Center for Violence and Injury Prevention at the George Warren Brown School of Social Work, the study was approved by the Human Research Protection Office at Washington University School of Medicine (WUSM), and all participating parents were individually consented to the study. The authors certify that the study was performed in accordance with the ethical standards as laid down in the 1964 Declaration of Helsinki and its later amendments.

## Results

### Stage 1: Results of HCC Validation

Hair cortisol concentration (HCC) exhibited marked temporal stability (ICC 0.70, *p* = 2.52e-05), greater than all other HPA-axis biomarkers measured, as depicted in the diagonal of Table 1, indicating that hair cortisol assays capture trait-like characteristics of HPA-axis variation in children in this age range. HCC was moderately correlated with PM salivary cortisol (*r* = 0.47 at baseline and 0.19 at three month follow-up) and uncorrelated with AM salivary cortisol (r =  − 0.01 at baseline and − 0.12 at follow-up). Both heart rate variability and PM salivary cortisol were moderately stable over the three month time window. These results reinforced the incorporation of HCC into the data analyses as the index of HPA-axis variation with the highest degree of temporal stability among the bioassays.

### Stage 2: Risk and Outcome in Early Head Start

Table 2 presents descriptive statistics of key characteristics of the sample at baseline. Of note, 29% (*N* = 39) of the mothers who completed a CES-D (*N* = 134) met the clinical cut-off for depression (as determined by a score of ≥ 16, per standard guidelines). CAN data were accessible for 79.7% of the 123 families (82.76% of 145 children); 50.8% of the 145 children lived in families with at least one Child Protective Services report of CAN.

A correlation matrix of selected quantitative risk and outcome indices employed in the study revealed that none significantly intercorrelated, except for the internalizing and externalizing scales of the CBCL (see Supplemental Table 1). Of note, neither hair nor salivary cortisol were significantly correlated with any of the other risk measures (nor with each other). Since child maltreatment has been demonstrated to incur additional risk for psychopathology, we analyzed presumptive measures of toxic stress as a function of official-report CAN (see Supplemental Table 2 for results of independent samples t-tests). No significant differences existed between groups (official-report maltreatment versus no maltreatment). Linear regression analysis failed to identify main effect predictors of child behavioral outcome as measured by the CBCL, as shown in Supplemental Table 3.

### Effects of TIY Pilot Intervention

#### Socialization Group Attendance

We examined attendance in three time blocks: (1) the 12 scheduled socialization groups prior to enrollment in BTS (“Pre”); (2) the pilot study interval during which 12 sessions of the TIY intervention were administered, along with continued socialization groups-as-usual for the controls (“During”); and (3) the 12 socialization groups following the randomized intervention (“Post”). The reported percentages are presented in Supplemental Fig. 1 reflect average attendance rate for the enrolled subjects. During TIY intervention interval, attendance at socialization groups was approximately three times higher in TIY groups than in TAU groups.

#### Child Abuse and Neglect

Among those *without* prior CAN reports, TIY group had 23.9% later reports compared to 27.9% of TAU; this difference failed to reach statistical significance, but exhibited a trend in the expected direction. It is important to note that among those *with* prior CAN reports, TIY group had 39.9% later reports compared to 60% of controls; however, there were only 5 controls, rendering our analysis without adequate power to detect a main effect. There was no greater likelihood of missing official report CAN data (not ever matched) between TIY and TAU groups. However, when limiting to children for whom we obtained a state match at baseline, there were more children whose family had prior official maltreatment reports among the TIY group than the control group ***χ***^**2**^ (1, *N* = 46) = 10.9, *p* = 0.001, suggesting that the intervention group may have been at higher risk at the time of enrollment.

#### Internalizing and Externalizing Behavior

We conducted repeated measures ANOVAs to ascertain if there was a change in CBCL scores between time points across TIY and TAU. There was no significant difference in CBCL score change between the groups. Pre- and post- descriptive statistics are outlined in Supplemental Table 4.

## Discussion

In this study involving measurement of key indices of putative toxic stressors among young impoverished children participating in EHS, we observed a lower-than-expected frequency of maternal-report childhood behavioral abnormalities and high rates of official-report child abuse and neglect (CAN). We also observed strikingly low associations between maternal depression, CAN, caregiver-child relationship quality, hair cortisol, and adverse child behavioral outcomes. In this study, TIY did not significantly augment effects of EHS in mitigating child psychopathology or CAN; there were trends for improvement, however, in the expected direction. Attrition rendered this analysis without sufficient power to derive a definitive determination of the intervention’s effectiveness, and despite randomization procedures the intervention group was found to be at higher risk at study onset than the control group.

Historically, CAN has been found to exert deleterious causal influences on child development resulting in enduring patterns of psychopathology (see Constantino, 2018; Jaffee et al., 2004; Jonson-Reid et al., 2010). The observation of relative typicality in the distribution of maternal report ratings of the children’s behavioral outcomes reflects opportunity for young children to withstand the effects of the substantial stressors of poverty (100% of the sample) and a history of official report CAN within the family (half of the sample) within the context of Early Head Start. The surprising nature of the lack of association extends beyond maternal ratings of their children’s behavior - predicted associations between objective indices of the parent–child relationship, CAN, HPA-axis variation, and maternal self-report of depressive symptomatology were similarly disrupted in this data collection despite the observed reliability of repeated measures. These unexpected findings are particularly important because of the uniqueness of the sample: it featured a within-cohort focus on young children who met rigorous standards for poverty; it incorporated official-report data on CAN which further documented a substantial level of environmental risk; and it was conducted in the context of a program (EHS) which is designed to provide intensive comprehensive child development and family support services to low-income infants and toddlers and their families, and therefore may have buffered the impact of this matrix of risk on the children’s outcomes.

The relative absence of hypothesized associations between the presumptive toxic stressors and between stress and child outcome may relate to a number of factors: (1) the impact of these presumed stressors in child development may have been attenuated in the context of EHS; (2) inferences about the associations of these variables from studies of lower risk samples may not hold true under conditions of extreme high risk; (3) the effects of these stressors on enduring psychopathology may manifest later in development; and (4) the maternal-report measures of child psychopathology may not have been sensitive or reliable enough to reflect early manifestations of psychopathology. What is clear from these data is that any one major risk factor (e.g., maternal depression) is not adequate to serve as a proxy for others, such as the measurement of parental emotional responsiveness or the occurrence of CAN, which has important implications for the ascertainment and interpretation of “risk” in future efforts in prevention science and policy. The other implication is that these findings identify important prospects for resilience of young children in the setting of what have been presumed to be toxic environmental stressors.

Limitations of the study include the following: (1) attrition over the course of the intervention, resulting in constraints on statistical power; (2) a low number of subjects in the clinical range of child psychopathology, thus reducing ability to ascertain associations between child psychopathology and risk and protective factors with small effect sizes; (3) absence of an epidemiologic sampling frame; (4) variable time periods families and children were enrolled in EHS prior to the start of our intervention, which may have attenuated relationships among risk variables and outcomes. We note that child behavioral abnormalities were ascertained by parent-report and therefore could have been systematically underestimated; however, the risk variables and their relationships with one another were predominantly objectively acquired. Finally, we note that there exist an array of possible parameters of resilience - at the level of child, family, and community - that may have disrupted expected relationships between risk variables in this study and that were unmeasured. These include genetic, family environmental, and social characteristics that promote adaptation in response to stress, the ability to specify which is evolving in child developmental science and will undoubtedly improve the ability to target intervention to children and families who can benefit from it most.

In this study, variation in HCC was not associated with the putative toxic stressors of maternal depression or official report CAN, nor did HCC predict adverse behavioral outcomes. Recent genetically informative studies have revealed that HCC is substantially heritable, around 72% (in line with our highly stable ICC estimates of HCC), and may more reliably index the effects of genotype than within-family environment - importantly, including socioeconomic status (Rietschel et al., 2016; Tucker-Drob et al., 2017). In a study of older twins, age 12–21 years, no significant phenotypic or genetic correlation existed between HCC and perceived stress, depressive symptoms, and neuroticism (Rietschel et al., 2017). Further, despite the measurement reliability of HCC, its usefulness in the present study may have been mitigated by the sample size and its complex relationships to other factors. It should also be noted that the elaboration of cortisol is only one facet of the complex cascade of biological responses to stress.

Testing theories about the biological effects of stress on development and psychopathological outcomes - particularly in humans - is challenging. These data indicate that caution should be exercised in drawing inferences about what constitutes “toxic stress,” especially when inferred from studies that are not designed to critically test causal associations and their potential confounds, one of which is unmeasured gene-environment correlation. Here, for example, a marker originally presumed to relate to toxic stress (HCC), and subsequently traced to genetic influences, proved to be minimally associated with major risks and outcomes related to overwhelming stress in early childhood. Moreover, the most notable finding of this study was that in a sample enriched for putative toxic stress factors (e.g., poverty, child maltreatment, maternal depression), the risk factors were only weakly correlated with one another, and the children were characterized by lower-than-expected levels of internalizing and externalizing psychopathology by their parents’ report. These findings suggest that our current understanding of “toxic stress” is under-developed. It is likely that young children vary in their degree of vulnerability to stressors associated with poverty, and that in this study of homogeneously impoverished families, unmeasured genetic factors may have disrupted the relationship between adversity and the outcomes we ascertained, reinforcing the need in future research to control for genetic variation (and its effects) across individual children in order to operationalize what constitutes “toxic stress.” A particular strength of this study is that it was conducted exclusively among imporverished families - often under-represented in child development and biomarker research - who were enrolled in the principle U.S. program designed to buffer the adverse consequences of poverty on early childhood development. More refined specification of salient deleterious exposures - as well as indices of inherited sensitivity and resilience - will improve opportunity for strategic targeting of preventive interventions to reduce enduring abnormalities in behavioral adaptation among infants and children at highest risk.

## Supplementary Information

Below is the link to the electronic supplementary material.

Supplementary file1 (DOCX 34 KB)
