# Supplementary material for: Parameterizing Toxic Stress in Early Childhood: Maternal Depression, Maltreatment, and HPA-Axis Variation in a Pilot Intervention Study
Source: Prev Sci. 2022 May 23;26(Suppl 1):78–89. doi: 10.1007/s11121-022-01366-4 (PMC12053303; doi:10.1007/s11121-022-01366-4)
Supplement: Supplementary file 1 — Supplementary file1 (DOCX 34 KB) [file 11121_2022_1366_MOESM1_ESM.docx]

Supplementary Materials: Text, Supplemental Tables 1-4, and Supplemental Figure 1

***Hair Cortisol Concentrations.***  using a commercially available enzyme immunoassay kit (Salimetrics, LLC) following a previously published protocol (Arch et al., 2014; D’Anna-Hernandez, Ross, Natvig, & Laudenslager, 2011; Hoffman, D’Anna‐Hernandez, Benitez, Ross, & Laudenslager, 2017). Cortisol concentration analysis and assay validation were conducted as previously described (Hoffman, D’Anna‐Hernandez, Benitez, Ross, & Laudenslager, 2017). Briefly, each hair sample was placed in a pre-weighed 2 ml cryovial (Wheaton, Millville, NJ, USA), washed three times in 100% isopropanol and dried. After washing, drying, and re-weighing samples on a high-sensitivity electronic balance (Mettler Toledo Model MS105, Greifense, Switzerland) to determine individual hair mass in these small samples, hair was ground in the same cryovial using a ball mill (Retsch, Haan, Germany) after adding a 4.76 mm carefully cleaned stainless steel ball bearing. Specially milled aluminum cassettes were designed to hold three cryovials. The cassettes, containing the cryovials, were submerged in liquid nitrogen for 3 to 6 minutes to freeze hair samples to facilitate grinding. Samples subsequently were ground for 4 to 5 minutes. Powdered hair was extracted in the same cryovial in 0.33-1.0 ml (depending on sample mass at a ratio of 5 pg hair/100 microliters (µl) methanol) high pressure liquid chromatography (HPLC) grade methanol for 24 hours at room temperature on a side-to-side shaker platform. Following methanol extraction, cryovials were spun for three minutes in a centrifuge at 15700g to pellet the hair. Then 133 µl of the extraction supernatant was removed, placed into a microcentrifuge tube, and dried under a stream of nitrogen in a drying rack in a fume hood at room temperature. The dried extracts were then reconstituted with assay diluent based on hair weight.

Cortisol levels were determined using a commercial high sensitivity Enzyme Immunoassay (EIA) kit (Salimetrics LLC, State College, PA, USA) per manufacturer’s protocol. Methods for assay cross validation with other laboratories using liquid chromatograph-mass spectrometry (LC/-MS/MS) and cross reactivity were described previously by (Russell et al., 2015). Cross validation entailed assaying 10 identical samples by four laboratories by EIA and/or LC/MS and comparing the resulting levels. Correlations across laboratories of r^2^>0.9 were noted for both EIA and LC/MS indicating excellent consistency and comparability across several laboratories. Intra- and inter-assay coefficients of variation were less than 10 and 5% respectively for all assays, regardless of cortisol matrix.

***Salivary Cortisol Concentrations (SCC).*** Saliva samples were assayed for cortisol concentration using a commercially available enzyme immunoassay kit (Salimetrics, LLC) following a previously published protocol (Arch et al., 2014). .

***Urine Cortisol.*** Urine samples were diluted in assay buffer and assayed for cortisol concentration using a commercially available enzyme immunoassay kit (Salimetrics, LLC) following the protocol described above.

**Supplemental Table 1: Correlation Matrix of Quantitative Measures**

|  | | | | | | | | | | | | | | | |
| --- | --- | --- | --- | --- | --- | --- | --- | --- | --- | --- | --- | --- | --- | --- | --- |
|  |  |  |  |  |  |  |  |  |  |  |  |  |  |  |  |
|  | |  | | **CCSERRS** | | **CES-D** | | **CBCL-I** | | **CBCL-E** | | **HCC** | | **SCC** | |
| CCSERRS |  | Pearson's r |  | — |  |  |  |  |  |  |  |  |  |  |  |
| CES-D |  | Pearson's r |  | 0.071 |  | **—** |  |  |  |  |  |  |  |  |  |
| CBCL-I |  | Pearson's r |  | 0.272 |  | 0.226 |  | **—** |  |  |  |  |  |  |  |
| CBCL-E |  | Pearson's r |  | 0.157 |  | 0.267 |  | **0.705** |  | **—** |  |  |  |  |  |
| HCC |  | Pearson's r |  | -0.06 |  | 0.03 |  | 0.06 |  | 0.04 |  | — |  |  |  |
| SCC |  | Pearson's r |  | 0.032 |  | 0.007 |  | 0.099 |  | -0.010 |  | 0.118 |  | — |  |
|  |  |  |  |  |  |  |  |  |  |  |  |  |  |  |  |

A matrix of Pearson two-tailed correlations between measures. *Note*: Bold values are statistically significant after Bonferroni correction for multiple comparisons.

| **Measure** | **Number of Subjects (No-CAN /CAN)** | **Mean (SD)** | ***t*** | **Degrees of Freedom** | **p-value** |
| --- | --- | --- | --- | --- | --- |
| **CES-D** | 58  61 | 11.89 (9.00)  14.18 (10.07) | -1.30 | 117 | 0.19 |
| **CBCL: Internalizing** | 40  38 | 41.5 (20.35)  40.39 (18.82) | 0.25 | 76 | 0.80 |
| **CBCL: Externalizing** | 40  38 | 42.15 (20.48)  40.89 (19.28) | 0.28 | 76 | 0.78 |
| **CCSERRS** | 56  59 | 17.11 (4.74)  17.54 (4.80) | -0.49 | 113 | 0.63 |
| **Hair Cortisol Concentration (HCC)** | 51  55 | 291.1 (1252.5)  581.5 (2528.5) | -.76* | 104 | 0.46 |

**Supplemental Table 2: Comparison of Measures as a Function of CAN**

Independent samples t-tests as a function of group (no-CAN vs. CAN). No-CAN includes only subjects for whom there was a documented absence of a report. *Satterthwaite due to folded F value.

**Supplemental Table 3: Linear Regressions of Maternal Depression and CAN on Psychopathology**

| **Dependent Variable: CBCL—Externalizing** | | | | | | | | | |
| --- | --- | --- | --- | --- | --- | --- | --- | --- | --- |
| **N** | **R^2^** | **Root MSE** | **Model F** | **Model *p-value*** | **Degrees of Freedom** | **Coefficient** | **β (SE)** | **t** | **Coefficient**  ***p-value*** |
| 77 | 0.039 | 19.8 | 1.30 | 0.278 | 2, 68 | CES-D | 0.32 (0.25) | 1.24 | 0.22 |
|  |  |  |  |  |  | CAN | -6.73 (5.05) | -1.33 | 0.19 |
| **Dependent Variable: CBCL—Internalizing** | | | | | | | | | |
| **N** | **R^2^** | **Root MSE** | **Model F** | **Model *p-value*** | **Degrees of Freedom** | **Coefficient** | **β (SE)** | **t** | **Coefficient**  ***p-value*** |
| 77 | 0.008 | 19.79 | 0.30 | 0.74 | 2, 68 | CES-D | 0.14 (0.25) | 0.57 | 0.57 |
|  |  |  |  |  |  | CAN | -3.30 (4.97) | -0.66 | 0.51 |

Independent variables included the following: maternal depression (CES-D) and any CAN, including prior CAN.

**Supplemental Table 4: Pre- and Post- Descriptive Statistics of TIY and TAU**

|  | **The Incredible Years** | | | | **Treatment at Usual** | | | |
| --- | --- | --- | --- | --- | --- | --- | --- | --- |
| **Measure** | **Baseline N** | **Baseline**  **Mean (SD)** | **FU N** | **FU**  **Mean (SD)** | **Baseline N** | **Baseline**  **Mean (SD)** | **FU N** | **FU**  **Mean (SD)** |
| **CBCL: Internalizing** | 62 | 40.11 (20.34) | 35 | 39.94 (15.75) | 28 | 41.14 (16.91) | 17 | 36.82 (19.89) |
| **CBCL: Externalizing** | 62 | 40.56 (20.54) | 35 | 39.54 (16.81) | 28 | 40.96 (17.25) | 17 | 35.47 (18.06) |

Baseline and follow-up descriptive statistics of CBCL scores for TIY and TAU groups.

**Supplemental Figure 1: Parental attendance at EHS group meetings prior to incorporation of TIY curriculum (pre), during the curriculum, and following completion of the curriculum (post).**
